# Differentiation of Pontine Infarction by Size

**DOI:** 10.1515/med-2020-0025

**Published:** 2020-03-08

**Authors:** Lei Yang, Wei Qin, Yue Li, Shuna Yang, Hua Gu, Wenli Hu

**Affiliations:** 1 Department of Neurology, Beijing Chaoyang Hospital, Capital Medical University, No. 8, South Gongti Road, Beijing 100020, China; 2 Department of Radiology, Beijing Chaoyang Hospital, Capital Medical University, Beijing China

**Keywords:** Lacunar infarction, Diagnosis, Pontine infarction, Branch diseas

## Abstract

**Purpose:**

We hypothesized that the current criteria may be unsuitable for lacunar pontine infarctions (LPI) diagnosis and that size criteria may indicate different stroke mechanisms.

**Methods:**

A total of 102 patients with isolated pontine infarctions were divided into a parent artery disease (PAD) and non-PAD groups according to stenosis of basilar artery. Further, 86 patients from the non-PAD group were divided into paramedian pontine infarction (PPI) and LPI groups. Data were collected from the three groups. The “golden” criterion for LPI was established based on the location of the infarction. A receiver operating characteristic (ROC) curve were used to evaluate the optimal cutoff value to use as an LPI diagnostic indicator.

**Results:**

There was a high prevalence of patients with PAD in both asymptomatic carotid atherosclerosis (ACAS) and PPI groups. Patients with PPI had a higher prevalence in diabetes and ACAS than those with LPI. Based upon the ROC curve, the optimal lesion size cutoff value for use as an LPI diagnostic indicator was 11.8 mm.

**Conclusions:**

Diffusion weighted imaging (DWI) cutoff points for predicting LPI may differ from that of the middle cerebral artery territory. The diameter of LPI may also indicate different stroke mechanisms.

## 1 Introduction

Lacunar infarction is an ischemic infarction of less than 15 mm in diameter located in the territory of the cerebral penetration arteriole. The most common topographies include the lenticular nucleus, thalamus, frontal lobe white matter, pons, basal ganglia, internal capsule and caudate nucleus. Fisher stated that hypertension is a specific etiology of lacunar infarcts, and segmental arterial disorganization and lipohyalinosis are the main pathological changes attributed to hypertension [1, 2].

The criterion of 15 mm lesion size originated from previous autopsy results; however, in an era of magnetic resonance imaging (MRI) technology, the current cutoff size criterion may have to be reconsidered. Sequence studies have also concluded that the 15 mm size criteria may no longer be useful for lacunar infarction [3, 4]. It is also worth noting that the 15 mm criterion for lacunar infarction includes anterior and posterior circulation. However, recent studies that attempted to challenge this standard have only focused on the middle cerebral artery (MCA) territory [3,4].

**Figure 1:**
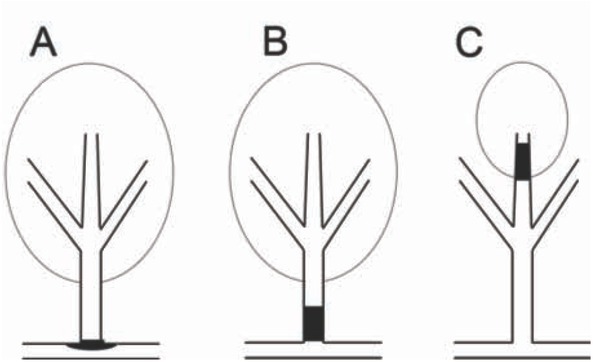
**Proposed mechanistic model of isolated pontine infarction**. **(A)** Paramedian pontine infarction (PPI) that results from plaque in the parent artery blocking the branch orifice. **(B)** Paramedian pontine infarction (PPI) that results from a microatheroma in the orifice of the branch. **(C)** Lacunar pontine infarction (LPI) that results from fibrinoid necrosis or lipohyalinosis of the distal perforating artery.

Isolated pontine infarctions are classified as either paramedian pontine infarctions (PPI) or lacunar pontine infarctions (LPI). It is widely accepted that PPI is caused by the occlusion of basilar perforating branches, whereas LPI is caused by small vessel disease (SVD) [5, 6]. Previous studies [7, 8, 9, 10, 11, 12] have shown that the infarction size of branch atheromatous disease (BAD) is larger in the MCA compared to the SVD; however, a research pertaining to the criterion such as size in the pons is rare. Our previous study revealed that lesion size is a useful method for distinguishing between SVD and BAD in the MCA territory (Figure 1) [13].

In this study, we aimed to determine whether size cutoff values for predicting lacunar stroke may be different for the anterior and posterior circulation. Further, we hypothesized that the 15 mm size criteria may no longer be useful for lacunar pontine infarction and that different size criteria may indicate different stroke mechanisms.

## 2 Methods

### 2.1 Study population

This retrospective study collected data from the records of consecutive patients admitted to the neurology department at the Beijing Chaoyang Hospital between January 2011 and December 2013 who had an isolated pontine infarction on diffusion weighted imaging (DWI).

Patients included in the study met the following criteria: (1) admitted within one week after stroke onset and (2) MRI showed isolated infarction in the pons. Patients with a definite cardioembolic source (e.g., atrial fibrillation, recent myocardial infarction, dilated cardiomyopathy, valvular heart disease, or infectious endocarditis) were excluded.

**Ethics Statement**: All subjects consented to participate in our study and signed an informed consent to the use of data for research. The design of this study was approved by the Ethics Committee of Beijing Chao-Yang Hospital, Capital Medical University.

### 2.2 Demographic and clinical assessments

All patients underwent laboratory testing for urea nitrogen (BUN), creatinine (Cr), uric acid (Ur), creatine kinase (CK), glycosylated hemoglobin (HbA1c), blood glucose (Glu), low density lipoprotein cholesterol (LDL), high density lipoprotein cholesterol (HDL), triglycerides (TG) and homocysteine (HCY). In addition, each patient received a cardiac evaluation that included an electrocardiogram and heart ultrasound.

Demographic features and risk factors were recorded, including hypertension (defined as receiving medication for hypertension or having blood pressure >140/90 mm Hg on repeated measurements), diabetes mellitus (defined as receiving medication for diabetes mellitus or diagnosed at discharge), hyperlipidemia (defined as receiving cholesterol-reducing agents or having LDL ≥2.6 mmol/L at the time of admission), current cigarette smoking, history of stroke and history of coronary heart disease (CAD). The National Institutes of Health Stroke Scale (NIHSS) score was measured at the time of admission and discharge.

### 2.3 MRI protocol and assessment

Brain magnetic resonance imaging (MRI) was performed within one week of onset of infarction, including DWI, fluid-attenuated inversion recovery (coronal) and magnetic resonance angiography (MRA). Brain MRI was performed with a 1.5T scanner (Signa Horizon LX, GE, American) or a 3.0T MR scanner (Siemens, Erlangen, Germany).

**Figure 2:**
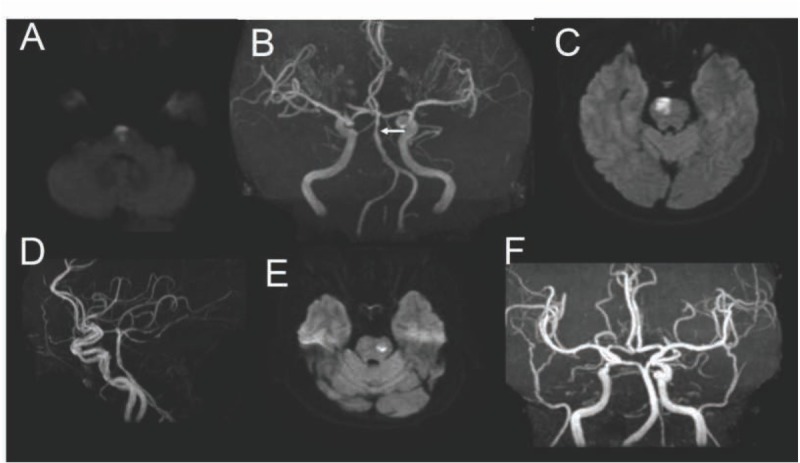
**Paramedian pontine infarct (PPI) and lacunar pontine infarct (LPI) in brain imaging of DWI and MRA image**. **(A)** Axial DWI showing a PPI. **(B)** MRA showing stenosis of the basilar artery of the same patient as A. **(C)** Axial DWI showing a PPI. **(D)** The basilar artery of the same patient as C is normal; **(E)** Axial DWI showing an LPI. **(F)** The basilar artery of the same patient as E is normal.

PPI was defined as a lesion extending to the anterior surface of the pons, and LPI was defined as a lesion that did not extend to the anterior surface of the pons (Figure 2). Parent artery disease (PAD) was defined as patients having stenosis of basilar artery. Concomitant asymptomatic cerebral arterial atherosclerosis (ACAS), defined as any extracranial and intracranial cerebral artery atherosclerotic disease unrelated to current isolated pontine infarctions, was also assessed. The diameter of the maximally involved infarct level (axial on DWI) was evaluated as a parameter for the size of the infarct.

### 2.4 Assessments of WMH

White matter hyperintensity (WMH) was assessed using a scale of periventricular hyperintensity (PVH) and deep white matter hyperintensity (DWMH), according to Fazekas scoring [14]. Two investigators (neurologists) independently reviewed the MRI and MRA images for each patient. In the case of a discrepancy, a third investigator (neuroradiologist) made the final decision.

Patients were then divided into two WMH burden groups based on their Fazekas scores as follows: ‘‘mild group’’, with a PVH/DWMH Fazekas score of 0 or 1, and ‘‘moderate-severe group’’, with a PVH/DWMH Fazekas score of 2 or 3.

### 2.5 Statistical analysis

Continuous variables with normal distribution are presented as means with standard deviations and were compared using the Independent-Samples T test, whereas variables with non-normal distribution are presented as medians with interquartile ranges and were compared using the Mann-Whitney U test. Categorical variables were compared using the Chi-square test. Receiver operating characteristic (ROC) curves were plotted to determine the optimal infarct size cutoff-points for predicting different types of pontine lacunar infarction. The “golden” criterion for lacunar infarction in the pons is defined as a lesion not extending to the anterior surface of the pons. We assessed discrimination by calculating the area under the ROC curve (AUC). An area of 1 implies a test with perfect sensitivity and specificity, while an area of 0.5 implies that the model’s predictions are no better than chance. Optimal cutoff-points for size were determined by maximizing the Youden index. Statistical analyses were performed using SPSS 20.0 software. P values of < 0.05 were considered statistically significant.

## 3 Results

### 3.1 Patient characteristics

During the study period, 102 patients had isolated pontine infarctions (69 males, 33 females) with a mean age of 64.9 ± 10.9 years (range, 41 to 91 years). Risk factors included hypertension in 75 patients (73.5%), diabetes in 49 patients (48%), cigarette smoking in 31 patients (30.4%), alcohol consumption in 41 patients (40.2%) and hyperlipidemia in 21 patients (20.6%). Additionally, 11 patients (10.8%) had histories of coronary heart disease. The median NIHSS score was 3 (interquartile range, 2 to 5). Sixteen patients (15.7%) had PAD and 82 patients (80.4%) had ACAS, while 72 patients (70.6%) had lesions extending to the surface of the pons.

### 3.2 Characteristics of isolated pontine infarctions according to PAD

Patients with PAD had a high prevalence of ACAS (P < 0.001) and PPI (P < 0.034). Conversely, there were no significant differences in the prevalence of male gender, hyper tension, DM, hyperlipidemia, history of CAD, smoking and alcohol consumption. There were also no significant differences in levels of glucose, CRP, ESR, DD, HCY, FBG, TG, TC and LDL-C (results are not listed).

**Table 1:** Characteristics due to the presence of parent artery disease (PAD)

Clinical features	Non-PAD N = 86	PAD N = 16	P valve
Age, years	64.9 ± 10.9	64.8 ± 9.4	0.971
Male	56 (65.1%)	10 (62.5%)	1
Hypertension	62 (72.1%)	13 (81.2%)	0.55
Diabetes	42 (48.8%)	7 (43.8%)	0.789
Hyperlipidemia	17 (19.8%)	4 (25%)	0.737
History of CAD	9 (10.5%)	2 (12.5%)	0.682
Smoking	28 (32.6%)	3 (18.8%)	0.379
Alcohol consumption	33 (38.4%)	8 (50%)	0.415
NIHSS (admission)	3 (2-5)	4 (2-6)	0.493
NIHSS (discharge)	2 (1-4)	4 (1-5)	0.345
PPI	57 (66.3%)	15 (93.8)	0.034*
ACAS	66 (77.6%)	16 (100)	0.037*
PVH (Fazekas) ≥ 2	42 (48.8 %)	6 (37.5%)	0.43
DWMH (Fazekas) ≥ 2	22 (25.6%)	5 (31.2%)	0.758
HbA1c (%)	7.6 ± 2.2	7.4 ± 1.6	0.706

Notes: Data are presented as mean ± standard deviation, median (interquartile range) or counts (%). CAD, coronary artery atherosclerosis disease; PAD, parent artery disease; PPI, paramedian pontine infarction; NIHSS, National Institutes of Health Stroke Scale; ACAS, asymptomatic cerebral arterial atherosclerosis; PVH, periventricular white matter hyperintensities; DWMH, deep white matter hyperintensities; HbA1c, glycosylated hemoglobin. *P < 0.05.

### 3.3 Characteristics of isolated pontine infarctions according to lesion type by DWI

Of 86 patients without PAD, 57 patients were PPI and 29 patients were LPI according to DWI. Patients with PPI had a higher prevalence of diabetes mellitus and ACAS than those with LPI. NIHSS scores were also higher in patients with PPI than in those with LPI.

**Table 2:** Characteristics according to lesion type by DWI in patients with normal basilar artery

Clinical features	LPI N = 29	PPI N = 57	P value
Age, years	64.2 ± 8.46	65.3 ± 12	0.616
Male	21 (72.4%)	35 (61.4%)	0.348
Hypertension	24 (82.8%)	38 (66.7%)	0.135
Diabetes	9 (31%)	33 (57.9%)	0.023*
Hyperlipidemia	8 (27.6%)	9 (15.8%)	0.253
History of CAD	2 (6.9%)	7 (12.3%)	0.712
Smoking	14 (48.3%)	14 (24.6%)	0.032*
Alcohol consumption	14 (48.3%)	19 (33.3%)	0.241
NIHSS (admission)	2 (1–3)	4 (3–6)	0.002*
NIHSS (discharge)	1 (0–2)	3 (2–6)	0.001*
Size of infarcts	7.03 ± 3.60	15.68 ± 3.76	< 0.001*
ACAS	16 (57.1%)	50 (87.7)	0.002*
PVH (Fazekas) ≥ 2	15 (51.7 %)	27 (47.4%)	0.82
DWMH (Fazekas) ≥ 2	8 (27.6%)	14 (24.6%)	0.797

Notes: Data are presented as mean ± standard deviation, median (interquartile range) or counts (%). LPI, lacunar pontine infarction; PPI, paramedian pontine infarction; CAD, coronary artery atherosclerosis disease; PAD, parent artery disease; NIHSS, National Institutes of Health Stroke Scale; ACAS, asymptomatic cerebral arterial atherosclerosis; PVH, periventricular white matter hyperintensities; DWMH, deep white matter hyperintensities; HbA1c, glycosylated hemoglobin. *P < 0.05.

### 3.4 Models for predicting PPI in patients with isolated pontine infarctions

**Figure 3:**
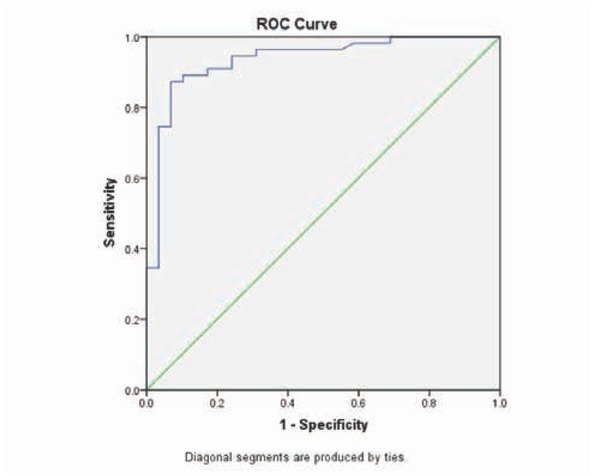
Receiver operating characteristic (ROC) curves evaluating the accuracy of using lesion size in LPI diagnosis.

Based on the ROC curve, the optimal lesion size cutoff value to use as an indicator for diagnosis of LPI was projected to be 11.8 mm (Youden index, 0.8038), which yielded a sensitivity of 87.3% and a specificity of 93.1%, with an area under the curve of 0.935 (95% CI 0.878–0.992; Figure 3).

## 4 Discussion

Our study showed that patients with PAD had a high prevalence of PPI (P < 0.034), and patients with PPI had a higher prevalence of diabetes mellitus and ACAS than those with LPI. Moreover, NIHSS scores were higher in patients with PPI than those with LPI. We also determined that PPI was the primary cause of isolated pontine infarction, suggesting that atherosclerotic perforating arterial disease is the most common cause of isolated pontine infarction.

Our findings are consistent with a study that included 67 acute infarcts involving the pons [5], which reported that patients with PPI have a significantly higher frequency of basilar artery stenosis and a worse prognosis than patients with LPI. Our results are also consistent with a study that looked at 101 isolated pontine infarctions [15], which reported that 79.2% of patients was PPI and BAD was the primary cause of IPI.

In 1965, Fisher reported his series of clinicopathological findings that formed the basis of the lacunar hypothesis, which established a relationship between focal symptoms, topography of lesion, etiology and therapy [1, 2, 16]. Hypertension was considered to be a specific etiology of lacunar infarcts, and lipohyalinosis was the main pathological change due to hypertension. Based on autopsy studies [1, 2], the maximal lesion diameter of 15 mm became a key criterion for imaging diagnosis.

In 1989, Caplan [6] proposed “intracranial b ranch atheromatous disease (BAD)” based on 3 autopsied cases with pontine infarcts. BAD was considered to be narrowing or occlusion of the mouth of the branching artery by an atheromatous process different from lipohyalinosis. He believed infarct of the pons extending to the basal surface resulted from BAD, while lacunar infarction in the pons that did not extend to the basal surface was due to lipohyalinosis.

From a pathological point of view SVD, also named hypertensive SVD, is mainly characterized by a loss of smooth muscle cells from the tunica media, deposits of fibro-hyaline material, and narrowing of the lumen and thickening of the vessel wall. This form of the disease is a common and systemic type that also affects the kidneys and retinas and is strongly associated with aging and hypertension [17]. However, pathological documentation of BAD has been rare. Based on a pathologic study of 3 patients, Caplan determined that either the orifices of the perforator artery could be blocked by an atheroma in the trunk, an atheroma could originate in the trunk and extend into the branch (called junctional atheromatous plaques), or microatheromas could arise at the origin of the branch itself [6].

SVD and BAD are difficult to directly visualize in vivo. Therefore, some markers of radiological phenotypes were selected as representatives for SVD, such as small, deep brain infarcts, cerebral white matter lesions, deep brain hemorrhages and cerebral microbleeds [17]. Further, diabetes mellitus, cerebral atherosclerosis and coronary heart disease may represent BAD.

In our study, patients with PAD had a high prevalence of ACAS and PPI. Patients with PPI had a higher prevalence of diabetes mellitus and ACAS than those with LPI. Also, both diabetes and coronary heart disease are more prevalent in patients with large artery atherosclerosis than in those with small vessel disease. Our results support that LPI is more closely related to SVD caused by lipohyalinosis, whereas PPI is more closely related to BAD caused by atherosclerosis.

A deep subcortical infarct in the perforating arterial territory with a diameter less than 15 mm is currently defined as a “lacunar infarct” due to small vessel occlusion. This concept, which is based on limited clinicopathological correlations, has always been controversial. Small (< 15 mm) subcortical infarcts can also be produced by internal carotid artery stenosis, middle cerebral artery (MCA) stenosis, artery-artery embolism or cardiac embolism (CE), while a larger infarct can also occur without evidence of cerebral vascular stenosis or CE.[18, 19]

The 15 mm criterion originated from previous autopsy results reflecting infarcts in the chronic stage, whereas DWI evaluations show acute cytotoxic or vasogenic edema. Therefore, in the era of MRI technology, this criterion should be reconsidered. Some studies [3, 7, 11] attempted to use a 20 mm diameter criterion for lacunar infarct. However, some studies were concerned that relying on an axial dimension of 15–20 mm may not be the best way to classify lacunar infarct [3, 4].

Research from Cho et al. [3] showed that there was no difference in clinical features or risk factors between an MCA stenosis group and an SVD group, or between SVD groups with lesions < 15 mm and ≥ 15 mm. Thus, they concluded that the 15 mm size criterion may no longer be useful for lacunar infarction. Bang et al. [4] found that a significant proportion of clinical MRI lacunae in patients were correlated with underlying non-lacunar mechanisms. Thus, the authors concluded that universal clinical MRI cutoff points for predicting lacunar stroke may not exist and that a grading system, rather than a dichotomizing one, is needed.

Neuroanatomical studies [20, 21] showed that the lenticulostriate arteries can only arise as individual vessels, by the use of common stems or in both ways. A recent pathological study [22] displayed first-order (proximal) to third-order (distal) branching of perforator arteries of the basal ganglia. Further, an MRI study [23] showed that the size of single small subcortical infarctions (SSSI) can be dependent on the branching order of the arteries involved, with first-order branches being associated with the largest infarct dimensions and the size of SSSIs reaching up to 30 mm. According to arterial anatomy, pontine perfusion territories can be categorized into four groups [24]: anteromedial, anterolateral, lateral and posterior. The anteromedial and anterolateral territories are supplied by basilar arterial branches.

We choose patients with isolated pontine infarction as the subject and evaluated infarct diameter cutoff points for predicting PPI or LPI. We also determined the “golden” criterion for lacunar infarction in the pons, which was defined as a lesion that does not extend to the anterior surface of the pons. This was different from previous studies using specific infarct size or lacunar syndrome, and we found that the lesion size is significantly bigger in PPI patients than in those in the LPI group. Based on the ROC curve, the optimal lesion size cutoff value for the diagnosis of PPI was projected to be 11.8 mm, which yielded a sensitivity of 87.3% and a specificity of 93.1%.

It is important for us to differentiate the two pathogeneses of lacunar infarction, small-vessel occlusion and branch atheromatous disease, since prognoses and treatment strategies differ between PPI and LPI. Patients with PPI had poorer prognosis than those with LPI. According to our study, the size of infarcts can help us distinguish different types of lacunar infarction. In the future, neuropathological examination of patients would be important for us to better understand cerebrovascular diseases [25].

Our study also had limitations. Firstly, we could not completely rule out whether isolated pontine infarctions were due to cardiac embolism or artery-to-artery embolism. Secondly, our study was a retrospectively hospital-based, and thus, selection bias was inevitable. Thirdly, basilar arteries were not examined with high-resolution MRI, so we could not exclude the presence of mild atherosclerotic plaques that did not cause significant luminal stenosis. Fourthly, our patients received 1.5 T or 3 T MRI, which may have increased the variability of the WMH assessment.

## 5 Conclusions

Our study has several novelties compared to previous studies. First, we proposed that clinical studies on lacunar infarction should separate the two blood supply regions. In addition, our research regarding lacunar infarction focused on the pons, which is different from the previous studies focusing on the territory of MCA. Lastly, in our study, we chose the “golden” criterion for location of infarction (extending or not to surface of pons), which differs from previous studies using specific infarct size or lacunar syndrome. DWI cutoff points for predicting lacunar stroke of pons may differ from territory of MCA.
